# Our Bureau of Information

**Published:** 1912-01-27

**Authors:** 


### OUR BUREAU OF INFORMATION.

### Homes for those of Moderate Means.

The developments of modern life tend to make certain forms of illness more common, and also to make it more difficult to treat these at home. In a flat—and so many people now live in flats—there is little space for a sick-room, and still less is there the possibility of maintaining the quiet that is necessary for all invalids, and especially for those who are suffering from the neurasthenia, which is a disease in itself and often accompanies other diseases. In cases which require the attendance of a nurse the problem is almost insoluble in the average town house of people of moderate means. The invalid has the spare bed-room, and there is none for the nurse. Doctors know that their patients would in many cases be cured sooner if they were removed to a nursing home, but they hesitate to recommend the removal because there is a general belief that nursing homes are exceedingly dear.

There is no alternative for those suffering from disease which necessitates a major operation, if they cannot pay the necessarily high charges of a home, but to go into a hospital—perhaps in a pay ward, but often as free patients. But where the requirements are simply care, suitable food and strict accuracy in the administration of medicine, a much simpler and less expensive home would be sufficient, and would prove a boon to many.

Sick-room cookery is not too familiar to the average servant, but a cook who knows about it can prepare food for a dozen as easily as for one. The visits of friends and relations should be limited to certain fitting times. Can these advantages be provided at a cost so little above that which illness involves in the patient's own home that it would not prevent their being accepted?

### Nursing Homes at Three Guineas a Week.

We think that it can be done. Nay, we are sure that there are many homes under the management of trained nurses and staffed with competent women, where patients could be received for about three guineas a week, perhaps even less in chronic cases. This is less than the cost of illness at home, if the services of a nurse are needed, and not greatly in excess even where members of the family do the nursing. Many doctors would be glad to know of such homes, and we believe that many nurses would find it advantageous to start them.

It must be understood that a cheap nursing home cannot be a striking house in a handsome street; but in every quarter of London, and of every other large town, good houses are to be had in side streets, or in roads from which fashion has departed, where there are large rooms and more generous accommodation than modern builders allow.

Such moderately-priced homes would be a boon also in the country and at the seaside. Again, one would not expect to meet them at a watering-place where rents are high, but there are many quiet healthy places where invalids and convalescents would be well, and which only want finding out. We should be glad to hear of such places in every part of the country; but it must be understood that they must meet all reasonable tests as to situation and management. It is not a question of luxury in appointments, but of cleanliness, sanitation, and careful management. It is desirable also that any one who wants to receive patients through our bureau should state exactly what class of cases are required—children or adults, chronic, mental, convalescent, or whatever it may be. We need hardly add that wherever possible a testimonial from a local doctor should be sent, as well as one from some other responsible person. A letter from a former patient will always carry weight.

These rules, of course, apply to all homes dealt with through the Bureau, and we mention them here simply because it must be understood that moderate terms are not to be regarded as an excuse for carelessness or inefficiency, though it is quite obvious that small charges must mean simplicity in all non-essentials.

### Needs and Helpers.

### Establishing a School Sanatorium.

"Loudon" writes:—What accommodation is required for a school sanatorium, and what is the fee charged?

[Wherever possible, it is better to have a separate cottage within the school grounds for a sanatorium. If you must have the sanatorium within the schoolhouse, an upper floor must be reserved for this purpose. Besides the sick ward proper, there ought to be an observation ward for cases which exhibit suspicious symptoms. Exclusive sanitary arrangements should be provided, a scullery with plenty of hot water for washing up, or at least disinfecting crockery, etc., and, of course, a bedroom for the nurse. In some schools a sanatorium fee—generally ten shillings per term—is charged to all pupils. In others the charge is made only when the pupil is ill, and varies according to his needs.]

### Training and Salary of Lady Dispensers.

"E. A. K." writes: Could you be good enough to inform me through your columns (1) what salary lady dispensers receive, and (2) what examinations they are required to pass?

(The salary of lady dispensers runs from £40 to £100 a year, the latter being exceptional. Three years' training is necessary, and to obtain a good post it is essential to pass the Pharmaceutical Minor examination. There is also, however, the examination of the Apothecaries' Hall, which is neither so difficult nor so expensive as the "Minor.")

### Our List of Approved Homes.

We are pleased to add to our list of approved homes the following: "The Haven," 6 Holly Park, Finchley, N. Principal, Miss Jessie Holmes, M.R.B.N.A.

"Mascot," Seaton, Devon. Principal, Nurse D. Mills. South aspect, quiet situation. All ordinary cases, also permanent, accouchement, and delicate children.

### Answers to Correspondents.

"Wymering."—We must have the lady's personal assurance that she wishes to have her home included in our list, in which case she must send testimonials from a doctor who has sent her cases, as well as from a patient or a patient's responsible friend.

"E. L. C.," "M. W.," "Buckstone," "F. W. G.," and "K. N.," will be answered next week.

### Rules for Correspondents.

Everyone who uses or wishes to help our Bureau of Information must be kind enough to observe the following rules:—

(1) Postal replies cannot be given. Exception will only be made in very special cases at the Editor's discretion.

(2) Queries or answers relating to different cases must be written, or preferably typed, on separate sheets of paper, and the writing must never be crossed.

(3) Questions and replies received not later than Wednesday will, as far as possible, be dealt with in our issue of the following Saturday week.

(4) Letters must have attached the name and address of the sender, with a pseudonym for publication if preferred.

(5) All applicants for insertion in our list of Approved Homes must send a registration fee of five shillings. The omission of this leads to delay and gives avoidable trouble.

(6) *All communications* for this department *must be accompanied by the coupon* to be found at the bottom of the third page of the cover on the inside, and should be addressed to the Editor of the Bureau of Information, c/o The Hospital, 29 Southampton Street, Strand, London, W.C.

